# An Insight into GPCR and G-Proteins as Cancer Drivers

**DOI:** 10.3390/cells10123288

**Published:** 2021-11-24

**Authors:** Preeti Kumari Chaudhary, Soochong Kim

**Affiliations:** Laboratory of Veterinary Pathology and Platelet Signaling, College of Veterinary Medicine, Chungbuk National University, Cheongju 28644, Korea; chaudharypreety11@gmail.com

**Keywords:** GPCR, G-protein, GPCR signaling, cancer

## Abstract

G-protein-coupled receptors (GPCRs) are the largest family of cell surface signaling receptors known to play a crucial role in various physiological functions, including tumor growth and metastasis. Various molecules such as hormones, lipids, peptides, and neurotransmitters activate GPCRs that enable the coupling of these receptors to highly specialized transducer proteins, called G-proteins, and initiate multiple signaling pathways. Integration of these intricate networks of signaling cascades leads to numerous biochemical responses involved in diverse pathophysiological activities, including cancer development. While several studies indicate the role of GPCRs in controlling various aspects of cancer progression such as tumor growth, invasion, migration, survival, and metastasis through its aberrant overexpression, mutations, or increased release of agonists, the explicit mechanisms of the involvement of GPCRs in cancer progression is still puzzling. This review provides an insight into the various responses mediated by GPCRs in the development of cancers, the molecular mechanisms involved and the novel pharmacological approaches currently preferred for the treatment of cancer. Thus, these findings extend the knowledge of GPCRs in cancer cells and help in the identification of therapeutics for cancer patients.

## 1. Introduction

GPCRs are the largest and most diverse group of membrane receptors that govern practically all physiological functions through G-protein signaling. As a result, GPCR dysregulation is related to a variety of human diseases and disorders, including type 2 diabetes [1], Alzheimer’s disease [2], hypertension [3], and heart failure [4]. According to a growing body of research, GPCRs, G proteins, and their downstream signaling targets have now been implicated in cancer initiation and development, where they can affect abnormal cell growth and survival. GPCRs also take part in tumor cell invasion and metastasis by activating Rho GTPases and causing cytoskeletal alterations, as well as angiogenesis, which supplies the cancerous mass with nutrients and provides avenues for metastasis. Finally, GPCRs aid in the creation and preservation of a favorable tumor microenvironment, with effects on nearby blood arteries, signaling molecules, and the extracellular matrix. Therefore, understanding the molecular relation between GPCRs and malignancies is very important as the pharmacological manipulation of these receptors will become increasingly desirable for the expansion of novel strategies to target tumor progression and metastasis.

GPCRs are known to modulate the processes such as proliferative signaling, replicative immortality, evasion of growth suppressors, resistance to apoptosis, initiation of angiogenesis, and activation of invasion and metastasis that are identified as the hallmarks of cancer [5]. There is sufficient evidence that suggests the role of GPCRs in the regulation of the maintenance, differentiation, and pluripotency of cancer stem cells [6]. Current drugs targeting GPCRs have shown excellent therapeutic benefits as GPCRs, like many other kinds of cell surface proteins, can be targetable in several malignancies. However, research into the involvement of GPCRs in cancer is directed towards certain GPCR members only. Massive efforts are presently ongoing to advance new GPCR-based drugs for cancer. Novel GPCRs that are changed in cancer have been discovered in genome-wide comprehensive investigations of different human malignancies, and they could be viable targets for cancer treatment development.

However, the significance of GPCRs in tumorigenesis to a great extent has been overlooked, despite the fact that GPCR dysregulation plays an important role in cancer. Limited information is found in relation to the profile of GPCRs expressed by cancerous cells. At this point, elucidating particular signaling cascades of “cancer driver” GPCRs along with optimal cancer-type-dependent activation of a functional GPCR along with the role of GPCR changes to tumor progression is critical. Additionally, to find effective targets for personalized treatment henceforth, it is critical to distinguish between cancer driver genes and non-participant genes.

In this review, we discuss a thorough overview of the role of GPCRs in cancers and their signaling mediators such as protease-activated receptors (PARs), chemokine receptors, Gα_12/13_ proteins, lysophosphatidic acid (LPA), GPCR-mediated signaling pathways including the Wingless and Int-1 (WNT) and Hippo signaling pathways, and the cross-talk between GPCRs and other receptors that can lead to signaling circuit transactivation. We also explore the emerging and potential therapeutic targets discovered and described in tumor biology.

## 2. GPCRs, GPCR Signaling, and Cross-Talk

GPCRs with around 900 representatives are the largest class of surface-bound receptors that control a variety of basic physiological processes, including growth, metabolism, and homeostasis [7]. GPCRs possess an extracellular N-terminus, followed by seven transmembrane (7-TM) α-helices (from TM-1 to TM-7) connected by three intracellular (from IL-1 to IL-3) and three extracellular loops (from EL-1 to EL-3), and finally an intracellular C-terminus. There are four primary types of GPCRs based on their pharmacological properties: Rhodopsin-like receptors are classified as Class A, secretin-like receptors are classified as Class B, metabotropic glutamate/pheromone receptors are classified as Class C, and frizzled receptors are classified as Class D. Among them, Class A is the most well-researched family with multiple members that play important roles in cancer biology, such as PARs, leucine-rich repeat-containing receptors (LGRs) including LGR5, a genuine stem cell marker for colon and breast tissues.

**Figure 1:**
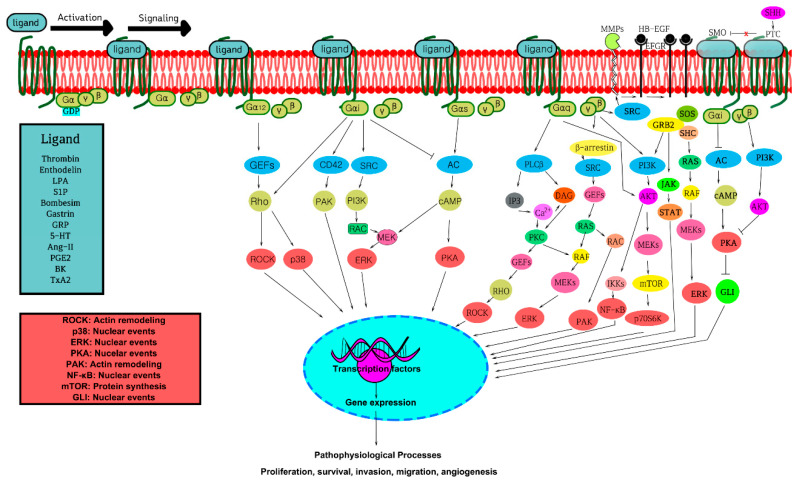
GPCR-mediated cell signaling pathways associated with cancer. Upon ligand binding, GPCR activates several downstream signaling pathways, including secondary such as GEFs for Rho, MAPKs, PI3Ks, along with their numerous cytosolic and nuclear targets. These receptor-mediated signaling cascades initiate various pathophysiological processes such as cell growth, survival, differentiation, tumor cell initiation, progression, and metastasis. Refer to the text for a detailed mechanism. Apdated from Lappano et al. [21].

GPCRs are linked to heterotrimeric G-proteins, Gα, Gβ, and Gγ, which in their natural condition bind the guanine nucleotide GDP. The Gα subunits are further classified into four classes: Gα_s_, Gα_i/o_, Gα_q/11_, and Gα_12/13_. Since the signal-transducing characteristics of the different possible βγ combinations do not appear to differ much, these classes are characterized by the isoform of their α-subunit. GTP displaces GDP-bound G-proteins once the GPCR is stimulated by ligand (hormones, lipids, peptides, and neurotransmitters) attachment to the extracellular N-terminus, enabling the dissociation of G-protein into a βγ dimer and a GTP-bound α monomer [7]. Although most GPCRs are capable of activating more than one Gα-subtype, GPCRs may also show a functional selectivity to one subtype over another, and the feedback pathways may result in receptor modifications (e.g., phosphorylation) that alter the G-protein preference. Because GPCRs are pleiotropic in terms of the cell signal proteins they activate, there are many conformations of the receptor that leads to a variety of highly specialized downstream signaling cascades (Figure 1). Essentially, there are two principal signaling pathways induced by GPCRs: the cAMP signal pathway and the phosphatidylinositol signal pathway [8]. Both Gα_s_ and Gα_i_ affect cAMP-generating enzyme adenylyl cyclase (AC). Gα_s_ stimulates AC, while Gα_i_ inhibits AC increasing or decreasing the cytosolic levels of cAMP, respectively [9,10]. Thus, a GPCR coupled to Gα_s_ counteracts the actions of a GPCR coupled to Gα_i_ and vice versa. Similarly, Gα_q_ activates phospholipase Cβ (PLCβ), which divides phosphatidylinositol 4,5-bisphosphate (PIP2) into diacylglycerol (DAG) and inositol 1,4,5-trisphosphate (IP3); DAG diffuses along the plasma-membrane and IP3 elevates the cytosolic calcium level [11]. These diffusible second messengers then target various ion channels, calcium-sensitive enzymes, and kinases such as cAMP-dependent kinase (PKA), protein kinase C (PKC), cGMP-dependent kinase (PKG), and calcium-calmodulin regulated kinases (CAMKs), which are further activated by cAMP, calcium/DG, cGMP, and calcium, enabling further biological effects (Figure 1). PKA regulates cell metabolism by phosphorylating particularly committed enzymes in the metabolic pathway, making it a key enzyme in cell metabolism. Gα_12/13_ regulate Rho family GTPase signaling through calcium-independent and Rho-dependent responses by activating RhoA-p160^ROCK^ pathways and are involved in the regulation of cell cytoskeleton remodeling. Certain GPCRs, such as the LPA receptors, can couple to several G-proteins, resulting in diverse signaling cascades, whereas others, for example, the sphingosine-1-phosphate receptor 1 (S1P1), can only couple to one G protein [12,13].

GPCRs have a distinguished role in cell migration, survival, and growth through the stimulation of multiple mitogen-activated protein kinases (MAPKs) cascades that include a family of greatly related serine/threonine kinases such as ERK1/2, JNK1-3, p38MAPKs, and ERK5, known to associate membrane receptors to transcription factors [14] (Figure 1). In addition, because of the role of GPCR-regulated MAPKs in gene expression, cell proliferation, and metastasis, specifically through Ras and Rho GTPases, MAPKs cascades have been studied in various pathological conditions, including human malignancies. Similarly, stimulation of the PI3K, AKT, and mTOR cascades and phosphorylation of multiple substrates have also been demonstrated to play a principal role in cell metabolism, migration, growth, and survival [15,16] (Figure 1).

GPCRs downstream signals cross-talk with integrin signals as well and transmit signals bidirectionally, so-called “inside-out” and “outside-in” signaling [17]. Following activation, integrins engage intracellular proteins involved in cytoskeletal reorganization and signal transmission by modulating the activation of tyrosine kinases such as focal adhesion kinases, Src, and PI3K, followed by activation of a cascade of kinases and small G-proteins of the Rho family, allowing numerous aspects of cell activity to be regulated. As a result, dysregulation of integrin function leads to a variety of diseases. In cancer, integrins play an important role in metastasis by promoting cell migration and invasion.

GPCRs undergo desensitization when exposed to their ligand for an extended amount of time by terminating the G-protein activation either via their own intrinsic hydrolysis capability often accelerated by RGS proteins or via PKA. In addition, GPCR may be desensitized itself through G-protein receptor kinases (GRKs)-mediated phosphorylation which enables arrestin recruitment (arrestin1 or arrestin2) that further terminates the downstream signaling, followed by subsequent internalization of the receptor into the endosomes. Finally, the internalized receptors are sorted either by degradation or recycling [18]. Several studies have been done that show the involvement of these GRKs and arrestins in cancer development and progression (Figure 1) [18,19,20,21].

## 3. GPCRs, G-Proteins, and GPCR Signaling Pathways in Oncogenicity

With the discovery of the MAS oncogene (the receptor for Angiotensin-(1–7)) in 1986, the direct relationship between cellular transformation and GPCRs was identified for the first time [22]. Since then, key GPCRs, their mutations, or changed expressions have been discovered by molecular genetics linking the GPCR family of proteins to tumor development and metastasis. GPCRs have been demonstrated to be implicated in cancer cell proliferation when triggered by an influx of a locally generated or circulating agonist. These agonists play a crucial function in angiogenesis and metastasis, and inflammation-related cancer. Previous studies also examined the discrete GPCRs in regards to expression, signaling, and functional activities of cancer [21,22,23,24]. It was demonstrated that a specific type of cancer cell/tumor expresses a common collection of GPCRs. Certain examples of a diverse group of GPCRs overexpressed in various primary and metastatic tumor cells and associated with tumor-cell growth when activated by circulating or locally produced ligands are shown in Table 1.

GPCRs are expressed by a large number of cells in the tumor microenvironment, in addition to cancer cells. Intercellular communication has been related to some GPCRs, such as chemokine receptors, which can help cancer cell proliferation, resistance to apoptosis, and other malignant phenotypic characteristics (Figure 1).

### 3.1. Aberrant Expression, Mutations, and Activation of GPCRs and G-Proteins in Cancer

Tumor cells, including those derived from various tissues such as lung, prostate, colon, pancreas, and mesenchyma, express GPCRs in an abnormal manner, including those GPCRs that drive cell proliferation, migration, invasiveness, and angiogenesis. Polymorphisms in the melanocortin-1 receptor, for example, have been linked to a higher risk of skin cancer [65]. Abnormal GPCR activation has previously been related to cell transformation, proliferation, angiogenesis, metastasis, and drug resistance due to high amounts of ligands such as LPA, D-erythro-S1P, and chemokines [21,66]. Furthermore, angiotensin II (Ang-II) and bradykinin (BK) receptors are overexpressed in LNCap and PC3 prostate cancer cells and drive cell proliferation via G_q_ and G_13_ signaling. Ang-II has the ability to stimulate androgen receptor (AR) expression in prostate cancer cells via the angiotensin-II type-1 receptor (AT1R) [67]. In PANC-1 pancreatic cancer cells, Ang-II and BK have been shown to speed up DNA synthesis [68]. The PI3K-Akt-mTOR cascade is also important for tumor cell proliferation, survival, migration, and metabolism [16,69]. Furthermore, GPR56, an orphan GPCR, can bind to G_12/13_ and activate Rho-dependent signaling pathways, increasing neural progenitor cell migration [70]. Human histamine receptor H1 (HRH1) is found in a range of malignancies, such as bladder, brain, blood, head and neck, lung, ovary, and skin [71]. The gonadotropin-releasing hormone (GnRH) receptor was found to be overexpressed in a variety of cancer cells, including melanoma, prostate, and endometrial carcinomas, leiomyomas, breast cancer, choriocarcinoma, and ovarian tumors [72,73,74]. The GnRH receptor can activate the G_i_ pathway in uterine leiomyosarcoma, as well as ovarian and endometrial carcinomas, resulting in down-regulated gene transcription and anti-proliferative effects in cancer cells [74]. GPR30 overexpression is linked to a worse survival rate in patients with endometrial or ovarian cancer, and it is also linked to an increased chance of generating metastases in patients with breast cancer [75,76,77].

Similarly, recent wide-scale sequencing attempts have revealed a plethora of mutations in GPCR genes associated with various human illnesses, along with cancer (Table 2). Clinical research paired with in vitro functional-expression experiments has found more than 600 inactivating mutations and nearly 100 activating mutations in GPCRs, which have been linked to over 30 human illnesses. Recent cancer genome mutation analyses have revealed that GPCRs are mutated in roughly 20% of all malignancies, including mutations in the thyroid hormone receptor (TSHR), the luteinizing hormone receptor (LHCGR), and follicle-stimulating hormone receptor (FSHR) in breast, lung, and colon cancers. Smoothened (SMO) that is negatively controlled by the twelve-transmembrane receptor Patched (PTCH) is one of the most commonly altered GPCR in malignancies [78,79]. PTCH and SMO mutations have been associated with the onset of sporadic basal cell carcinoma [80,81]. SMO is also mutated in malignancies of the colon and central nervous system, among other places. Squamous non-small cell lung cancer (NSCLC), adenocarcinomas, and melanomas have all been linked to mutations in the glutamate receptors GRM8, GRM1, and GRM3 [82].

Similarly, many GPCRs, including the arginine vasopressin receptor 2 (AVPR2) [83,84,85], RHO [86,87], and Melanocortin 2 receptor (MC2R) [88,89,90,91], have mutations that causes disease (Table 2). More research is needed to completely comprehend the molecular repercussions of these alterations, as well as their long-term effects on tumor growth. 

**Table 2:** Lists of activating/inactivating mutations of GPCRs in various cancers.

Receptor (IUPHAR)	Mutations (Amino Acid Changes)	Associations	References
Thyroid-stimulating hormone receptor (TSH receptor)	N-terminal: S281I; ICL3: D619G; A623V; L629F; TM6: F631L; T632I; D633H; ECL2: I568T; ECL3: V656F	a. Activating mutations; b. All mutants activating the cAMP pathway; c. Found in human thyroid carcinoma, breast, lung, and colon cancers	[92,93]
Melanocortin 1 receptor (MC1R)	TM2: D84E; TM7: D294H	a. Activating mutations; b. Related with human melanoma and nonmelanoma skin cancers.	[94]
	ICL2: R151C; R160W	a. Inactivating mutations; b. Changed the relative risk of nomelanoma skin cancer.	[91]
Melanocortin 2 receptor (MC2R)	R137W; S74I; Y254C	a. Activating mutations; b. Involved in adenoma and carcinomas.	[95]
Lutropin (LHCG) receptor	TM3: L457R; TM6: D578H; C581R; TM6: A572V; D578Y	a. Activating mutations; b. Found in human Leydig-cell tumor.	[96,97]
Smoothened (SMO) receptor	N-terminal: R199W; TM6: D473H; TM7: S533N; W535L; C-terminal: R562Q	a. Activating mutations; b. Found in human sporadic basal cell carcinoma (BCCs), lung and colon, and central nervous system cancers.	[98,99]
Follicle-stimulating hormone receptor (FSHR)	ECL2: D576G/N; TM4: D581G/Y; C584R; TM6: H615Y; D619G; A623I/S/V	a. Activating mutations; b. Slightly increasing in basal cAMP production; c. Found in human large intestine cancers, colon.	[100]
Brain-specific angiogenesis inhibitors 1–3 (BAI1–BAI3)	BAI1: N-terminal: S927A/D BAI3: GPS domain: G586R; C819Y TSP domain: T420I; A442E; W461L; 7TM domain: A1024P; R1050K; R1124C; C1148F; M1258I; F1378Y; G1404V; N1475T; D1449E; P1510L	a. Activating mutations; b. Found in human squamous lung carcinoma and lung adenocarcinoma.	[101,102]
EGF LAG seven-pass (CELSR1–3)	CL1: T838A/P (Gain domain) CL1 and CL3: K561N; D798H; V696L; A760Q; S810L; E811Q (Gain domain)	a. Activating mutations; b. All mutants are especially found in human squamous lung carcinoma and lung adenocarcinoma.	[102,103]
Latrophilins (LPHN)	LPHN1: A73D;V696L LPHN2: Q693H LPHN3: H18R; N344I; T442N; K561N; A760G; D798H	a. All mutants were activating mutations; b. All mutants were involved in tumor angiogenesis, invasion, or tumor growth.	[104]
Glutamate family of G protein-linked receptors (GRM1–8)	GRM3: N-terminal: G475D; G561E; S610L(ECL1); E767K(ECL2); E870K(C-terminal) GRM8: N-terminal: G49R; L76M; T118I; V150I; W215C; A282D; G523W; S691T; A808M (ECL3)	a. Activating mutations; b. GRM3 is mutated in 7% of human non-small cell lung cancer adenocarcinoma; c. GRM3 mutants are found in human melanoma cancers. a. Activating mutations; b. GRM8 is mutated in 8% of human squamous non-small cell lung cancer and melanoma cancers.	[102,105]
Muscarinic receptor	M1: TM2: F77I; TM3: W101A; TM6: E360A; Y381A; ICL3: K362A; N-terminal: I211A; Y212A M3: ECL2: Q207A; C257A; C264A	a. Activating mutations; b. Inactivating mutations; c. M1 mutants are found in human melanoma cancers. a. Inactivating mutations; b. M3 mutants are found in human melanoma cancers.	[106]
Lysophosphatidic acid receptor (LPAR)	LPAR1: ICL2: R163W; ICL3: R241Q L LPAR2: ICL2: R146H; ICL3: P230L LPAR3: ICL3: K216A; V219A; TM6: A247V LPAR4: R232H(ICL3) LPAR6: TM4: S154A; TM6: N248Y; TM7: L277P	a. Activating mutations; b. LPAR1 was mutated in human lung, neuroblastoma, and liver cancers. a. Activating mutations; b. LPAR2 was mutated in human colon cancers; a. Activating mutations; b. LPAR3 was mutated in human melanoma cells and osteosarcoma cells. a. Activating mutations; b. LPAR6 was mutated in human melanocarcinoma.	[107,108]
Sphingosine 1-phosphate (S1P) receptor	S1PR1: N-terminal: R13G; TM3: R120P; ICL3: T236A; R231K; R233K	a. Inactivating mutations; b. Involved in tumor growth, invasion and metastasis; c. Found in human lung, breast, and prostate cancer.	[109]

TM—transmembrane α-helix; ECL—extracellular loop; ICL—intracellular loop; C-terminal—Carboxy-terminal cytoplasmic tail; N-terminal—N terminus extracellular; GPS domain—G-protein receptor proteolytic domain; TSP domain—thrombospondin 1 domain; Gain-domain—GPCR autoproteolysis inducing domain.

G-proteins also play an important role in cancer and as cancer drivers due to their mutations (for details, please refer to [110]). Briefly, the Gα_q_ family encoded by *GNAQ*, *GNA11*, *GNA14*, and *GNA15* transmit many mitogenic signals upon GPCR stimulation [31,111]. A study with Gα_q_ subunit mutants induced malignant transformation in NIH3T3 cells, which were found to be tumorigenic in nude mice [112]. The majority of ocular melanomas have mutations in *GNAQ* or *GNA11*, and 6% of cutaneous melanomas have also shown mutations in these genes. *GNAQ* and *GNA11* are found to be significantly mutated in uveal melanomas, where they act as driver oncogenes by activation of JNK, p38, and AP-1-mediated transcription [113,114,115,116,117]. Surprisingly, this signaling circuitry was unaffected by PLC, Gα_q_’s most well-known target, and resulted in the activation of YAP, a transcriptional coactivator controlled by the Hippo pathway. In around 10% of skin cutaneous melanomas, the Gα_q_ family is also mutated. Mutations in Gα_q/11_ at residue R183 are the second most commonly mutated site in *GNAQ* that have been to cause ipsilateral occipital leptomeningeal angiomas, and sometimes uveal melanomas [116]. Activating Gα_q_ mutations have also been linked to congenital hemangiomas, as well as a group of other melanocytic neoplasms such as blue nevi, Ota nevi, and primary melanocytic tumors of the central nervous system [115,116]. Loss of Gα_q_ expression or recurrent loss-of-function mutations at T96S or Y101 are seen in around 25% of natural killer (NK)/T cell lymphoma, a malignant and highly aggressive subtype of non-lymphoma Hodgkin’s. The proclivity of R183 and Q209 hotspot mutations in Gα_q_ to solid tumors compared to T96 and Y101 in hematopoietic malignancies suggests an important interrelationship between the oncogenic or tumor-suppressive role of these mutations and the cell context in which they originate, emphasizing the complicated molecular events underlying Gα_q_-driven oncogenic signaling.

*GNAS*, which codes for the Gα_s_ protein, is one of the most frequently altered G proteins in various cancer such as appendix cancers (70%), pituitary tumors (27%), endometrial carcinomas (7.3%), stomach adenocarcinomas (5.7%), adrenocortical carcinomas (5.5%), pancreatic adenocarcinoma (5.6%), esophageal carcinomas (4.9%) and colorectal cancers (4.7%) [118]. Activating mutations in *GNAS* were demonstrated to induce endocrine cell hyperplasia, with activating mutations found in 28% of growth hormone-secreting pituitary tumors and 5% of thyroid adenomas [119,120]. *GNAS* mutations have been found in 4.4% of various malignancies [82]. The hotspot of the majority of Gα_s_ mutations occurs at R201 and Q227 [121]. *GNAL*, also encoding for Gα_s_, is shown to be mutated in nearly 7% of adrenocortical carcinomas and most pancreatic adenocarcinoma. These genetic alterations and autocrine activation play a critical role in the deregulation and activation of the PKA, Wnt, and MAPK pathways. Interestingly, many Gα_s_ mutant tumors (mostly gastro-intestinal neoplasms) have been discovered to be extremely mucinous. In contrast to the pro-oncogenic effects of Gain of function mutations in Gα_s_, Gα_s_ have been reported to drive tumor initiation and progression through de-repression of the Sonic Hedgehog and Hippo pathways in certain stem-like cell states [122,123,124]. In epidermal and hair follicle progenitor cell populations, conditional deletion of Gα_s_ causes fast development of basal cell carcinoma by repressing PKA-mediated inhibition of SHH and YAP signaling [125].

The Gα_i/o_ subfamily of G-proteins signals via a variety of effectors, including MAPK and PI3K activation. Constitutively active mutants of Gα_i_, like other G-proteins, have been found to have the ability to convert cells and are called proto-oncogenes [126,127]. When cancer-derived activated mutations of *GNAO1*, which encodes Gα_o_, are produced in cells, they induce oncogenic transformation and anchorage-dependent growth [128]. Inactivating mutations in Gα_i/o_-coupled receptors are mutually exclusive with activating mutations in Gα_s_, implying that they have the same functional effects, meaning enhanced cAMP activity. Indeed, the Gα_i/o_ subfamily of G proteins is altered at a similar frequency in GI malignancies as the Gα_s_ subfamily; however, the functional importance of these mutations has not yet been thoroughly explored [129]. Upregulated cAMP/PKA activity may be a common cause of carcinogenesis in different tissue types, which needs additional exploration, both from a signaling and clinical standpoint, given the recurring occurrence of Gα_s_ pathway activation in GI malignancies.

*GNA12* and *GNA13*, jointly known as the gep oncogene, encode two subunits that make up the Gα_12_ subfamily of G-proteins. Wilt-type Gα_12_, the only G protein subfamily whose overexpression is sufficient to be transformative without mutation, was discovered in a sarcoma-derived cDNA library screen to promote the transformation of NIH3T3 cells [130]. Gα_12/13_ can communicate with a variety of effectors, including catenin, radixin, and MAPK [131,132,133,134]. These signaling pathways control a wide range of cancer-related transcriptional networks and cellular processes, including AP-1, STAT3, and YAP activation. Overexpression or mutation of Gα_12/13_ or Gα_12/13_-linked GPCRs such as PAR1 or thromboxane A2 receptor (TBXA2R) has been proven to be transformative and considerably boost the invasive potential of many cancer types, including breast, prostate, and hepatocellular carcinomas [30,118,135,136,137]. Consistently, blockade of Gα_12_ signaling has been reported to considerably diminish the metastatic capacity of 4T1 mouse breast cancer cells and significantly enhance the metastasis-free lifespan of mice in breast cancer mouse models [135]. Interestingly, in a variety of hematological and lymphoid cancers, including Burkitt’s lymphoma and DLBCL, the Gα_13_ /RhoA signaling axis has been revealed to have a tumor-suppressive effect [138,139,140]. Upstream effectors, such as the Gα_12/13_ linked Sphingosine-1-phosphate receptor-2 (S1PR2) and P2RY8, a suspected Gα_12/13_-coupling orphan GPCR, and downstream effectors, such as ARHGEF1, have also been reported to contain mutations [141,142,143]. The mechanism by which the inactivated Gα_13_ signaling pathway increases lymphoma formation is unknown; nevertheless, multiple investigations have indicated that suppressing the G_13_/RhoA axis causes an increase in phosphorylated-AKT in B cells [141]. Targeting the PI3K/AKT pathway may be a potential therapeutic strategy for patients with Gα_13_ deletion, since raised pAKT levels may be observed in immunohistochemistry of DLBCL tumors, and high pAKT is linked with poor survival in DLBCL patients [144].

By modifying or potentiating G-protein-driven signaling, functional participants of G-protein signaling, including RGS family proteins and βγ subunits of the heterotrimeric G-protein, can have pro-oncogenic effects [145]. Recent pan-cancer investigations have identified transcriptome dysregulation and hundreds of mutations in RGS proteins, enriched for those leading to LOF, boosting G-protein activity via a hitherto unknown tumor-suppressive function and method of G-protein signaling potentiation [146,147]. RGS7, for example, is often mutated in 13% of melanomas, promoting anchorage-independent growth, migration, and invasion [148]. In breast and bladder malignancies, a near homolog, RGS6, has been discovered to have tumor-suppressive activities [149,150]. Gβγ subunits have also been discovered to be involved in cell migration and metastasis. The expression of Gβγ mutant in breast cancer cells were shown to greatly reduce extravasation, matrix breakdown, and macrophage-stimulated tumor cell invasion, indicating that Gβγ may have a role in paracrine signaling between tumor and immune cells [151,152].

In conclusion, both mutation and aberrant expressions are biological factors that lead to GPCRs and heterotrimeric G-proteins losing their normal function and gaining pro-oncogenic capacities. Further research into the link between various cancers and the functional duality of GPCRs, G-proteins, and signaling pathways is expected to uncover previously unknown cancer-causing processes, as well as novel treatment targets.

### 3.2. GPCR-β-Arrestin Signaling in Cancer

β-arrestin recruitment is associated with desensitization of GPCR-mediated signaling and promotes clathrin-dependent-endocytosis of activated GPCR. β-arrestins have been linked to a variety of outcomes, operating as multifunctional scaffold proteins and signaling transducers that are important for intracellular signal transmission and amplification, as well as controlling other cellular consequences. As a result, through diverse signaling pathways such as Src/MAPK; Wnt; Hedgehog; NF-κB and PI3K/AKT, β-arrestin1 and β-arrestin2 play various roles in the regulation and progression of malignant tumors [19,153]. β-arrestin1 has been shown to act as a scaffold for cytoskeleton remodeling in tumor cell motility [154,155,156]. Among the noncanonical activities of β-arrestin1, several studies have shown that nuclear β-arrestin1 may coordinate transcriptional responses to environmental perturbations, revealing new roles for β-arrestin1 in tumor growth [157,158,159,160]. Recently, a clinical study proposed β-arrestin2 as an important prognostic factor and also a promising target for new therapeutic approaches in advanced ovarian cancer [161].

A variety of β-arrestin-biased ligands, which cause preferential activation of the β-arrestin pathway over the G-protein-mediated signaling, have been identified, including EP2- and EP4-receptors and endothelin type A ETARs [162]. It was demonstrated that silencing the effects of β-arrestins in ETAR signaling decreases Src-EGFR-mediated transcriptional activity preventing β-arrestin-mediated ovarian cancer cell invasion and metastasis [163]. Similarly, very recently, a study using breast cancer cells demonstrated that reducing the expression of β-arrestin1 and β-arrestin2 tended to increase cell proliferation and invasion, whereas increasing their expression levels inhibited them [164]. β-arrestins have been shown to serve opposite roles in the development of lung and hepatocellular cancer [165,166]. In prostate cancer, β-arrestin2 inhibits cell viability and proliferation by repressing AR signaling [167,168]. Other findings, on the other hand, support the idea that β-arrestin2 action aids in the development of human tumors; β-arrestin2 is overexpressed in a variety of human tumors, including breast and renal cell carcinoma, and correlates with advanced stage and poor patient survival; and β-arrestin2 mediates a variety of tumor-promoting effects, including cell migration and invasion [169,170,171,172]. The anti- and pro-cancer actions of β-arrestins in various cancers may be influenced by the tumor microenvironment.

Furthermore, because β-arrestin-biased signaling necessitates phosphorylation of GPCRs by GRKs to promote high-affinity binding of β-arrestin to GPCRs, and because GRK subtypes may have preferential phosphorylation and trigger unique conformational changes in GPCRs, studies of β-arrestin-biased signaling may also consider the role of GRKs in cancer-related signaling pathways [173]. As stated in a recent study [174], various isoforms of GRKs can affect the response to several GPCRs implicated in tumoral signaling via direct interactions with other components of transduction cascades. As a result, GRKs are important in controlling the destiny of β-arrestin-dependent GPCR signaling and as prospective cancer therapy targets.

In conclusion, β-arrestins integrate GPCR signals with intrinsic cellular pathways, starting intracellular signaling waves in a G protein-independent manner and permitting the identification of novel therapeutics targeting selectively β-arrestin-mediated circuits known as biased arrestin-biased agonism [175]. Therefore, anticancer and tumor suppressor effectiveness of β-arrestin isoforms that elucidate their function specialization should be examined further to fully understand the mechanisms underlying the role of β-arrestins in cancer.

### 3.3. Biased Agonism towards Specific G-Proteins in Cancer

A signature characteristic of a biased GPCR ligand is the capability to activate either of the G-protein subtypes (Gα_s_, Gα_q/11_, Gα_i/o_, or Gα_12/13_) for selectively mobilizing and exploiting specifically selected GPCR-mediated downstream signaling pathway in various metabolic disease systems, including cancer [176]. In regards to the involvement of G-proteins in cancer, while the majority of G-proteins are not linked to cancer, the Gα_12/13_ family has been linked to cell transformation (e.g., fibroblasts) [177,178], pointing to tumor-related mechanisms. Migration, proliferation, transformation, platelet aggregation, neurite retraction, and actin-stress fiber production are just a few of the cellular activities regulated by Gα_12/13_ sub-family proteins [26,179,180]. GPCR ligands including thrombin, LPA, and S1P, for example, stimulate tumor growth and invasion by specifically tying their corresponding receptors to Gα_12/13_ proteins, suggesting that Gα_12/13_, PARs, LPA, and S1P receptors are a significant issue in cancer progression. The stimulation of the Rho-dependent pathway regulates cytoskeletal dynamics, transcriptional regulation, cell cycle progression, and cell survival, which are considered a major contributor to cancer initiation and progression in the Gα_12/13_ sub-family of G-proteins.

Likewise, Gα_q/11_ and Gα_i_ are also shown to be selectively linked to LPA receptors. The LPA3 receptor is shown to be connected to Gα_i_ in NIH 3T3 and neuroblastoma B103 cells, resulting in Ras-GTP buildup of MAPK activation and increased cell proliferation [181,182]. LPA1, LPA2, and LPA3 receptors in PC12 cells are found to be linked to Gα_q/11_ following neurokinin A or endothelin binding, initiating signaling via tyrosine kinase c-Src [49,50,183].

Identically, G-proteins have been shown to direct biased agonism in various metabolic diseases other than cancer. For example, following bias ligand activation, GPR109A couples to Gα_i/o_ to induce levels of high-density lipoprotein and decrease triacylglycerol levels leading to the prominent decrease in cardiovascular morbidity and mortality [184]. N-linked glycosylation of PAR1 at EL2 favors coupling to Gα_12/13_-dependent Rho activation, while EL2 with no glycosylation favors Gα_q_-coupled phosphoinositide signaling [185]. Biased agonism has the potential to activate not just distinct G protein subtypes but also an alternative signaling mechanism, such as β-arrestins, which may mediate positive effects rather than receptor internalization and degradation activating and scaffolding the cytoplasmic signaling complexes [176].

In contrast to the successful adoption of the GPCR biased signaling idea for therapeutic benefit in the cardiovascular, neurological, and behavior sectors, there have been no publications establishing the efficacy of GPCR biased signaling for the treatment of cancer. Recent research, however, revealed scientific advances in the possible application of biased signaling on endothelin receptors in cancer therapy. Endothelin-A receptor (ETAR) couples to Gα_q_, Gα_s_, and Gα_12/13_ and is expressed mainly in vascular smooth muscle cells and cardiomyocytes as well as solid tumors [186,187,188]. The activation of the ETAR by endothelin-1 (ET-1) is a key factor in the development of ovarian cancer by promoting anti-apoptosis, invasion, and neoangiogenesis [189]. Indeed, overexpression of ETAR is linked to a poor prognosis in patients with ovarian carcinoma. However, a clinical trial found that particular ETAR antagonists are no effective as a cancer therapy adjunct. This might be due to ETAR’s signaling bias, which controls both oncogenic and tumor-suppressive activities. Gα_q_-coupled or β-arrestin-dependent signaling pathways are known to mediate ETAR’s carcinogenic downstream effects [49,189]. The recruitment and nuclear translocation of β-arrestin, which in turn works as an epigenetic regulator of multiple angiogenic/metastatic genes, including β-catenin, is facilitated by GRK5/6-mediated phosphorylation of the receptor [49,190]. ETAR-mediated Gα_s_ activation, on the other hand, stimulates AC/cAMP/PKA signaling, which has been shown to limit tumor growth in numerous carcinoma-derived cell lines [17,191,192]. Because the ET-1/ETAR axis may activate both tumor suppressive and oncogenic features in cancer cells, ligands that target Gα_s_/cAMP/PKA signaling could be a promising new treatment option for a variety of cancers. Silencing both β-arrestin1 and β-arrestin2 also reduces the signaling of these receptors (e.g., ETAR), lowering Src and serine/threonine kinase AKT activation, and ultimately altering the β-catenin pathway [163]. CXCR4 overexpression and dysfunctional downstream signaling have been linked to tumor development, vascularization, and metastasis in a variety of malignancies [193]. PAR2, a GPCR with unique biased signaling, has also emerged as a possible therapeutic target for preventing breast cancer cells from quickly metastasizing [194]. As a result, the creation of novel biased ligands for CXCR4 and PAR2 might lead to new cancer therapy options.

### 3.4. GPCRs in the Hallmarks of Cancer

#### 3.4.1. GPCRs in Migration, Invasion, and Metastasis

Metastasis, or the migration of tumor cells via blood or lymphatic arteries to other organs, is one of the most critical difficulties in cancer treatment [195]. Cancer cells are known to selectively metastasis to specific organs rather than spread randomly, with a higher prevalence [196]. It is known that chemokines can guide cell movement by causing changes in the cytoskeletal structure and dynamics of receptor-bearing cells, thus, enabling metastasis. Additionally, chemokine production locally in the tumor microenvironment attracts macrophages and leukocytes that enhance the cytokine-rich milieu and cause the secretion of matrix metalloproteases (MMPs), which help in the cancer cells survival, proliferation, and invasion. Moreover, GPCRs of chemokine receptors have been shown to be crucially connected to organ-specific metastasis in various malignancies. It has been shown that tumor cells with abnormal chemokine GPCR expression co-opt chemokine migratory activity, enabling metastasis to various organs [197].

CXCR4 is one of the most well-known chemokine receptors with proliferative, survival, and migration effects that are shown to be aberrantly expressed in many cancers and are involved in metastasis. The most common sites of metastasis, such as lymph nodes, lungs, bone marrow, and liver, express CXCL12/SDF-1, which is a chemokine ligand for CXCR4 [196]. It has been shown that CXCR4 is abundantly expressed in breast cancer cells. Its stimulation activates Rac1 via P-REX1 that is involved in most breast-type cancers’ metastasis. CXCR4 may also couple to G_12/13_, promoting metastasis in a RhoA-dependent manner in basal-like breast cancer cells [198]. Therefore, targeting either molecule engaged in the control of CXCR4 expression on tumor cells or the downstream signaling could provide therapeutic options.

Additional chemokine receptors, such as CCR7 and CCR10, were shown to directly take part in cancer cell survival and proliferation as well as metastatic homing [199]. Furthermore, several new studies are currently being conducted to learn more about the adhesion family of GPCRs and their possible roles in cancer development and metastasis [200]. Recently, it was revealed that GPR116, a member of the poorly understood adhesion GPCR family, has a role in the invasion and migration of breast cancer cells by activating the Gα_q_-RhoA-Rac1 pathway.

#### 3.4.2. GPCRs in Tumor-Induced Angiogenesis

Very recently, Nag et al. reviewed the several aspects of cardinal GPCRs that are involved in tumor angiogenesis [201]. Tumors release angiogenic factors that promote endothelial cell migration and proliferation, inducing the development of new capillaries following the increased demands of tumor cells’ food and oxygen. Many angiogenic agents, such as thrombin, prostaglandins, S1P, and chemokines, operate on GPCRs expressed on endothelial cells [202,203,204]. Some chemokines, such as CCL2, CCL5, and CXCL8/IL-8, attract leukocytes and macrophages to the tumor site, where they can release vascular endothelial growth factor (VEGF) and other angiogenic factors that help new blood vessels form [203]. Furthermore, inflammatory cytokines secreted in the tumor microenvironment enhance COX-2 expression and local release of prostaglandin E2 (PGE2), which boosts tumor and stromal cell expression of proangiogenic VEGF, CXCL8, and CXCL5 [205].

GPCRs and their ligands can induce angiogenesis either directly by increasing endothelial cell proliferation or indirectly by boosting the release of VEGF and other angiogenic factors from stromal, immune, or malignant cells. Tumor vascularization supplies nutrients for tumor expansion as well as invasion and metastasis routes.

#### 3.4.3. Inflammation and Immune Cell Evasion in Tumor Microenvironment

The association between PG synthesis and tumor progression is one of the better acknowledged of the several mediators linking inflammation and cancer. The cyclooxygenases COX-1 and COX-2 produce PGs, and the binding of PGs to their corresponding GPCRs expressed in numerous cells initiates their pro-inflammatory actions. Nonsteroidal anti-inflammatory medications (NSAIDs) that inhibit COX-1/2 have been demonstrated to lessen the risk and incidence of a variety of cancers [206,207]. COX-2 inhibition with NSAIDs, for example, lowers the overall incidence and size of adenomas in patients genetically susceptible to colorectal cancer and is an effective chemopreventive therapy in healthy people [206,207].

PGE2 and signaling through its associated GPCRs, EP1–EP4, have been widely studied in relation to tumor growth [208,209,210]. EP1 couples to Gα_q_, but EP2 and EP4, which are more important in colon cancer, couple to Gα_s_ that promote cAMP buildup [208]. PGE2 can promote various signaling pathways in colon cancer cells, including β-catenin [211,212] and the nuclear hormone receptor peroxisome proliferator-activated receptor δ (PPARδ).

Chemokines can also attract macrophages to a tumor’s location. The involvement of CCL2 in the recruitment of CCR2-bearing tumor-associated macrophages (TAMs), which play critical roles in tumor vascularization and development, has been widely explored. CCL5 has been connected to macrophage recruitment in the past [197,213]. Some immune cells, on the other hand, can aid in the destruction of tumor cells; in this situation, the tumor chemokine microenvironment may aid in evading the immune surveillance system by triggering a less effective humoral response while blocking cell-mediated immune responses to tumor cells [197,213].

#### 3.4.4. Tumor-Suppressor Functions of Some GPCRs

Mutation of certain GPCRs and G proteins might act as a tumor-suppressor gene in some cancers. For example, inactivating mutations in the MC1R have been shown to elevate the chance of developing melanoma [214]. CXCR3 ligands have been demonstrated to decrease tumor advancement by indirectly mediating anti-angiogenic effects, while the cannabinoid receptors CB1 and CB2 have been shown to suppress tumor progression in a variety of malignancies, including gliomas, breast, colorectal, and skin cancers [215]. In diffuse large B cell lymphoma (DLBCL), SIP2 receptor signaling via G_α13_ may also have tumor-suppressive effects [216]. Although G_α13_ signaling has a role in tumor progression and metastasis, lower expression or inactivating mutations in S1P2 and/or G_α13_ may actually promote tumor progression in DLBCL. Similarly, the GPR54/KiSS1-derived peptide receptor has been shown to limit metastasis in melanoma and breast cancer cells [217]. There are likely to be many more GPCR-G-protein signaling pathways that need to be uncovered henceforth that could have anti-tumorigenic effects in various malignancies.

## 4. Cancer-Associated GPCR-Mediated Signaling Pathways

There are a diverse group of GPCRs-mediated signaling pathways involved in a variety of primary and metastatic tumor cells and link themselves to cancerous growth when activated by circulating or locally produced ligands. Some of these signaling pathways are briefly described below.

### 4.1. Wnt Signaling

Wnt proteins play a crucial role in malignant events like cancer in addition to physiological development, and tissue homeostasis and its signaling pathway mediated by Frizzled (Fz) receptor (also called Wnt receptors) has been the theme of vigorous research. The canonical Wnt signaling pathway stabilizes β-catenin by antagonizing the β-catenin “destruction complex” composed of Axin, adenomatis polyposis coli (APC), glycogen synthase kinase3 (GSK3), casein kinase1 (CK1), and the E3 ubiquitin ligase component TrCP1 via Fz-lipoprotein-related protein 5/6 (LRP5/6) receptor complex. The “destruction complex” continuously degrades the major effector of this pathway, β-catenin, in the absence of Wnt. Once stabilized, β-catenin is translocated to the nucleus of the cell and is implicated in the regulation of cell differentiation and proliferation. Some other GPCRs that actively participate in the β-catenin stabilization path include PTHR1, prostaglandin receptors, LPA receptors (LPA1–6), and endothelin receptors (ET1–4). Hyperactive stabilized β-catenin is found in a variety of malignancies, either as a result of oncogenic mutations in its N-terminal phosphorylation site or as a result of mutational inactivation of its negative regulators APC or Axin [218,219]. Activated β-catenin has the potential for carcinogenicity, especially in colorectal, breast, lung, oral, cervical, and hematopoietic malignancy. Additionally, Wnt signaling enhances its effect on tumorigenesis by influencing the tumor microenvironment via fine cross-talk between altered cells and invading immune cells, such as leukocytes. Wnt signaling also plays a role in epithelial-mesenchymal transition (EMT), thereby promoting the maintenance of cancer stem cells (CSCs).

Noncanonical Wnt signaling, which is also transduced by Fz receptors, does not use the LRP5/6 co-receptor and does not involve β-catenin/Tcf activity. Wnt5A/B, for example, are prototypes for this Wnt pathway [220]. Noncanonical Wnt signaling is implicated in planar cell polarity (PCP), dorsoventral patterning, tissue regeneration, convergent extension movements, and cancer in vertebrates. The noncanonical signaling mediates the Rho-associated kinase (ROCK) pathway, one of the key cytoskeleton regulators, and in general, opposes canonical Wnt/β-catenin signaling. Another example of β-catenin-independent signaling is the Wnt-Ca^2+^ pathway that regulates the nuclear factor of activated T cells (NFAT) and TAK1-induced Nemo-like Kinase (NLK) and is implicated in cancer development.

Tumor microenvironment and the growth factors secreted by stromal cells of the tumor microenvironment play a role in Wnt/β-catenin signaling. For example, stimulation of hepatocyte growth factor in colorectal cancer cells has been known to promote phosphorylation of β-catenin in tyrosine residue and its dissociation from Met via the PI3K pathway enabling tumor growth and invasion [221]. Similarly, PDGF, EGF, and TGF-β phosphorylated p68 promoted translocation of β-catenin initiating EMT [222]. Wnt/β-catenin signaling promoted VEGF-dependent angiogenesis in mouse models [223].

The precise role of G proteins in Fz-mediated Wnt/β-catenin signaling is an intriguing yet unsolved element. While some studies have indicated that G proteins influence Wnt signaling [224,225,226], other investigations have failed to identify G proteins as a critical element of Wnt/β-catenin signaling [227,228]. A MEF cells transfection study demonstrated a lack of interaction between Gα_i_ and the Wnt/β-catenin pathway [228]. The study showed that in the presence of exogenous Wnt3a, G proteins are not sufficient to promote Wnt/β-catenin signaling in MEF cells; nonetheless, they have diverse actions in modifying Wnt/β-catenin signaling. Gα_s_ enhances Wnt/β-catenin signaling, whereas Gα_q_ and Gα_13_ reduce it, and Gα_i_ has no impact under the identical experimental settings. G proteins should be necessary for Wnt/β-catenin signaling in all cell types if they are key aspects of Wnt/β-catenin signaling. By contrast, the authors concluded that Gα proteins were not part of the core Wnt/β-catenin signaling pathway and are not generally required for pathway transduction [228]. As a result, the role of G proteins in Wnt signaling pathways is still a hotly debated topic.

### 4.2. Hippo Signaling Pathway

The Hippo-Yes-associated protein (YAP)/transcriptional coactivator with PDZ-binding motif (TAZ) pathway are considered oncoproteins and have come out as a key preserved system that controls cell growth and transformation, organ size, mechanical and cytoskeletal proteins, polarity, and cell adhesion [229,230]. Dysregulation of this system leads to the development of cancer. YAP and its homolog protein TAZ, two essential downstream effectors of Hippo signaling, are important constituents in cancer. Thereby, scientists are working to produce pharmacological inhibitors of both YAP and TAZ, which are important targets for tumor drugs. The tumor-suppressing Hippo pathway is involved in limiting YAP/TAZ nuclear localization and transcriptional activity, and when the Hippo system is disrupted, the oncogenic YAP pathway is activated. YAP/TAZ are dislodged from their cytoplasmic anchoring site and translocate to cell nuclei after the Hippo enzymatic cascade is blocked. They act as transcription coactivators in the nucleus, stimulating downstream target genes and, as a result, promoting oncogenicity via binding to TEAD family transcription factors. The Hippo pathway’s Mst1-2-Lats1/2 kinase cascade suppresses YAP/TAZ via direct phosphorylation, resulting in cytoplasmic retention via 14-3-3 binding, which promotes -TrCP-mediated YAP/TAZ ubiquitination and destruction. GPCRs were discovered to be effective inducers of the YAP oncogenic pathway during the quest for physiological YAP/TAZ activators following the initial discovery of S1P- and LPA-YAP/TAZ activity [231,232,233]. GPCRs implicated in cell proliferation have been shown to stimulate the coactivator YAP’s transcriptional activity [229,233,234,235,236]. GPCRs have been shown to regulate the Hippo pathway differentially. LPA and thrombin receptors-mediated Gα_12/13_, Gα_q_, or Gα_i_ pathways activate YAP/TAZ while epinephrine and glucagon receptors-mediated Gα_s_ pathway inhibits YAP/TAZ. GPCRs have been demonstrated to decrease LATS activity via Gα_12/13_, thereby freeing YAP from LATS-dependent repression [233]. Oncogenic mutations in Gαq activate YAP via a mechano-sensing pathway and actin polymerization, rather than through interference in the Hippo-suppressing pathway, according to research from the Gutkind group [237]. PKA is thought to mediate upstream signals by inhibiting actin fiber production or directly phosphorylating LATS1/2 [238,239,240]. PKC appears to have isoform-specific effects; for example, classical PKC isoforms promote YAP/TAZ activity, whereas novel PKC isoforms suppress it [241]. MST1/2 does not appear to be a direct target of GPCR signaling; however, MAP4Ks-mediated LATS1/2 phosphorylation is responsive to diverse GPCR ligands [231,242]. Therefore, protein kinases (such as PKA and PKC), Rho GTPases, and actin cytoskeleton remodeling are most likely involved in the activity of GPCRs and G proteins in Hippo signaling in a tissue-dependent manner, but the mechanism remains still unclear [243]. Furthermore, new research has identified YAP/TAZ as genuine downstream effectors of the noncanonical Wnt signaling pathway that includes Wnt-FZD/ROR G_12/13_-Rho GTPases-Lats1/2, thus, increasing oncogenic YAP/TAZ- and TEAD-mediated gene transcription stimulation [244]. Hedgehog (Hh) ligands also cause YAP/TAZ suppression via the SMO-Gαs-cAMP-PKA signaling axis [122]. These findings suggest the involvement of atypical GPCRs (FZD, SMO) in the regulation of the Hippo pathway and cross-talk between the Hippo and other crucial pathways in cancer development. Additionally, when insulin is present, the influence of GPCR on YAP/TAZ activity mediated through PI3K and PKD downstream of the insulin receptor has been shown to be amplified [245]. The Hippo pathway has also been demonstrated to be modulated by MAPK signaling [246]. Some studies refer to YAP1 as a Wnt/β-catenin target gene. It has been demonstrated that β-catenin/TCF4 complexes directly regulate YAP gene expression, increasing its expression. Others have shown that TAZ interacts with DVL and thereby inhibits Wnt 3A-induced β-catenin stabilization. Hence, future research should focus on the cross-talk between the GPCR-Hippo signaling axis and other pathways. Furthermore, aberrant GPCR signaling might be a factor in the widespread activation of YAP/TAZ in human malignancies and demands detailed investigation.

Weakening YAP and/or TAZ can be a reasonable procedure for the treatment and prevention of a broad range of malignancies, given that induced transcriptional activities of YAP/TAZ are prominently engaged in cancer. Reduced YAP dose by shRNA depletion could be one strategy. A comprehensive panel of human cancer cell lines was examined for shRNA-induced mortality, and it was discovered that cancer cell lines stimulated for WNT signaling are particularly vulnerable to YAP knockdown [247]. As a result, inhibition of YAP is not always correlated with YAP activity, and that YAP inhibition may entail crucial TEAD-independent YAP-mediated interactions that are important for some cancer cells. Recently, in in vitro and in vivo, verteporfin (VP) and VGLL4-mimicking peptides have been utilized to inhibit YAP/TAZ activity, tissue growth, and cancer, however additional development of these medicines may be necessary for therapeutic usage. Furthermore, as the Hippo pathway is critically regulated by GPCR-mediated downstream signaling, drugs targeting GPCRs and G proteins may reduce YAP/TAZ activation and delay cancer progression. Gα_s_-targeted compounds, for example, may suppress YAP/TAZ activity in a similar fashion to epinephrine, dobutamine, and glucagon [248,249]. Antagonizing or reducing Gα_12/13_-, Gα_q/11_-, or Gα_i/o_-mediated signals, and using phosphatase-resistant LPA analogs and monoclonal antibodies selective for LPA or S1P [250,251], might reduce YAP/TAZ function. FR900359, a cyclic depsipeptide, has recently been demonstrated to bind mutant Gα_q_ and suppress MAPK and YAP downstream effectors [252,253]. Forskolin or phosphodiesterase inhibitors like Rolipram have been reported to activate PKA and suppress YAP/TAZ [122,250]. PKC inhibitors can also suppress YAP/TAZ activity depending on the cell type [254]. Statins, inhibitors of HMG-CoA reductase (HMGCR), have been found to indirectly inactivate Rho GTPases and diminish YAP/TAZ nuclear localization, which is important for the control of the Hippo pathway via GPCR signaling [255,256]. However, certain GPCR-based medicines, such as blockers and dopamine, have been shown to have considerable impacts on cardiac and psychological functioning; therefore, negative consequences must be addressed before employing these medications in cancer therapy [257,258]. 

### 4.3. PARs and Cancer

Proteinases and their inhibitors [259] make up nearly 2% of all human genes. While proteases regulate tissue functions through both non-receptor and receptor-mediated methods, their presence in the genome demonstrates their importance in controlling a wide range of tissue functions. The proteolytic enzymes such as thrombin and trypsin, like traditional growth factors, epidermal growth factor, and insulin, are able to initiate cell proliferation through activation of membrane receptor PARs [260,261,262,263]. There are four types of PARs: PAR1/2/3/4, which are activated by cleavage of part of their extracellular domain [264].

PAR1, the family’s original and most famous member, mediates the signaling in response to thrombin in nearly all cell types while PAR3 and PAR4 operate as a “back-up” mechanism for PAR1 [265,266,267,268]. PAR2 is triggered by a trypsin serine-protease as well as proteases found upstream of thrombin [269]. PARs are activated by enzymatic digestion of the N-terminal extracellular region, which results in newly exposed ligands that operate as signal transmitters via intramolecular attachment to extracellular loop number two [270].

It has been reported that PARs play an important role in oncogenesis including metastasis, and angiogenesis [271]. PAR1 has been shown to induce bone metastasis in prostate cancer, motility of colon carcinoma cells, and cell proliferation in melanoma. Interestingly, a study showed that PAR1 activation is not sufficient and requires co-activation with PAR2 agonist to induce migration and metastasis in melanoma [272]. PAR2 alone, or PAR3 and PAR4 agonists used alone or with PAR1, has no effect on metastasis, indicating that PAR2 regulates thrombin-dependent tumor cell migration and metastasis. Similarly, it was reported that co-activation of PAR1 and PAR2 contributes to vascular smooth muscle cells hyperplasia leading to restenosis (PAR2 modulates PAR1-driven neointimal hyperplasia), suggesting the importance of PAR1 and PAR2 in cancer. PAR1 and PAR2 were found to be highly expressed in clinical patients with esophageal carcinoma [273]. PAR1 and PAR2 both contribute to melanoma cell migration [272], breast cancer development [274,275], and cell proliferation and migration in colon cancer [276]. A study also reported that PAR2, rather than PAR1, signaling promotes the development of mammary adenocarcinoma in polyoma middle T mice [277]. In contrast, in most tumor cells, PAR4 functions as a tumor suppressor. The up-regulation of PAR4 has been known to induce apoptosis in prostate cancer cells [278], and decreased expression of PAR4 results in aggressive gastric cancer [279], breast cancer recurrence, and poor prognosis [280,281], and the promotion of colon cancer cells [282]. Although PARs are expressed in tumor cells and in the cells of the tumor microenvironment, the exact underlying signaling mechanism remains unknown. PAR1-RhoA pathway leading to cell rounding, disruption of intercellular junctions, cytoskeletal reorganization regulates cancer metastasis. Unlike PAR1, PAR2-dependent reorganization of the actin cytoskeleton, pseudopodia formation, and chemotaxis is mediated through the activation of Rac/p21-β-arrestin-ERK1/2 pathways and may be implicated in cancer migration and metastasis. Similarly, while PAR1 induces cellular activity in tumor cells via α_V_ integrins, in M24met melanoma cells, PAR2 has been shown to mediate migration via α_5_β_1_-dependent downstream signaling transduction molecules [272] The binding of signal proteins with a pleckstrin-homology (PH)-domain such as AKT (lipid-dependent binding), Etk/Bmx (lipid-independent binding) and Vav3 to signal-associating motifs in C-tails of PAR1 and PAR2 has been demonstrated to be critical for breast cancer progression [283]. PAR1 has been shown to enhance migration of a squamous cell carcinoma cell line and rat smooth muscle cells by trans-activating tyrosine kinase receptors such as epidermal growth-like growth factor via up-regulation of a matrix metalloproteinase [284,285]. Additionally, activation of PAR1 and PAR2 has been suggested to induce hematogenous metastasis as circulating tumor cells generate thrombin. PAR2 being directly activated by tissue factor (TF) has also been shown to play a role in tumor angiogenesis and growth. TF VIIa (FVIIa) also activates PAR2 that regulates proangiogenic growth factor expression as well as cross-talk with integrins via upregulation of VEGF through MAPK signaling [286], thus adding the crucial role of PARs and its signaling in cancer. Later, Schaffner et al. demonstrated a cross-talk of tumor cell TF cytoplasmic domain and PAR2 signaling and showed that TF domain has additional roles via recognized β-arrestin recruitment site that are interdependent with PAR2 signaling in regulating host angiogenic responses in a TF and PAR2-positive clinical breast cancer [274]. A study also showed that PAR2 agonists facilitate breast cancer cell chemokinesis through the Gα_i_-c-Src-JNK-paxillin signaling pathway [287]. Recently, Lidfeldt et al. provided a novel insight into the respective role of PAR1 and PAR2 in human breast cancer by showing that PAR2 was confined to the estrogen receptor (ER)-positive sub-group and PAR2 was an independent prognostic factor specifically in ER-positive tumors, while PAR1 correlated with worse prognosis specifically with ER-negative group [288]. Nevertheless, PAR signaling is also known to prevent apoptosis and thus, may also contribute to cancer progression. PARs are also activated by alternative pathways that lead to tumor cell proliferation, migration, invasion, metastasis as well as angiogenesis [289]. These findings clearly indicate the importance of PARs in cancer progression.

It has been demonstrated that a point mutation in H349APAR2, but not in R352A, effectively reduces PH-protein binding and is enough to significantly reduce PAR2-induced breast cancer growth in vivo and extravillous trophoblast (EVT) invasion in vitro. In a similar manner, the PAR1 mutant hPar1-7A is also unable to associate with the PH domain and significantly reduces breast cancer progression and EVT invasion. Moreover, very recently, Grisaru-Granovsky et al. evaluated the impact of PAR1 and PAR2 on physiological EVT invasion for early placenta development by demonstrating that PAR2 is necessary and required for PAR1-induced β-catenin stabilization through the formation of PAR-LPR5/6-Axin complex, paralleling the Wnt signaling pathway in an independent manner [290]. These findings indicate the significance of PAR PH domain binding motifs in both pathological and normal invasion processes. The palmitoylation of a cysteine residue in the C-tail of PAR1 and 2 could be one rationale for membrane targeting.

In conclusion, PARs might be the potential biomarkers and very likely lead to the development of potent therapies against various cancers.

## 5. Key Individual GPCRs and Their Signaling Pathways Involved in Various Cancer

Various receptors, including GPCRs, activate various signaling pathways and cross-talk with other membrane receptors to stimulate crucial pathophysiological functions in normal and cancerous cells [291]. For example, acetylcholine muscarinic receptors (mAChRs), epidermal growth factor (EGFR), and platelet-derived growth factor (PDGFR) receptors cross-talk with each other to activate mitogenic pathways to regulate cell proliferation, differentiation, and survival. Similarly, various kinds of ligands can activate a single receptor that can induce stimulatory effects in various kinds of cancers. One such action has been reported in EGFR that is transactivated by a number of GPCR ligands, including BK, LPA, Gastrin-releasing peptide (GRP), and bombesin (BN) [21]. As GPCRs are able to interact with other cancer-related membrane receptors, targeting these receptors can have significant anticancer effects.

Some examples of GPCRs and downstream signaling pathways that have been shown to play an important role in cancer progression are briefly explained below.

### 5.1. GPR30

GPR30 mediate diverse physiological functions to estrogens in normal circumstances. GPR30 overexpression has been shown to be present in numerous cancers. GPR30 controls the progression of hormonally sensitive malignancies such as endometrial, ovarian, thyroid, prostate, lung, and breast cancer, according to a large body of evidence, and can reduce survival rates. It has been reported that GPR30 stimulates both fast signaling and transcriptional processes in response to estrogen stimulation [292]. GPR30 is involved in cell survival, migration, adhesion, and Ca^2+^ mobilization and relates to G_s_ and G_i/o_. GPR30 facilitates G_s_ activation, which in turn activates adenylyl cyclase, causing intracellular Ca^2+^ mobilization as well as the activation of MAPKs and PI3K [293]. Through the G_i/o_ protein, GPCR30 also causes fast, non-genomic estrogenic effects inducing the release of heparin-bound EGF (HB-EGF) and subsequent matrix metalloproteinase-dependent transactivation of EFGRs [294,295,296]. It has been found that GPR30-mediated EGFR-ERK1/2 signaling triggers growth arrest of estrogen receptor (ER)-positive breast cancer cells [297,298]. An in vivo finding revealed that G-1 therapy greatly slowed the growth of SkBr3 xenograft tumors and improved survival, strongly suggesting that GPR30 is a potential key target and G-1 could be a promising therapeutic candidate for the treatment of ER-positive breast cancer. Importantly, some clinical research projects have shown that 4-hydroxytamoxifen and ICI 182,780 induce GPR30-mediated activation of downstream signaling pathways involved in the regulation of target gene expression and increase cell proliferation in a variety of cancer cells [293,299,300,301,302]. These medicines are commonly applied in cancer therapy, but they can also be employed in vitro to illustrate the possible outcome of activated GPR30 [303]. Overall, these studies suggest that cross-talk between the GPR30 and EGFR signaling pathways may be important in cancer medication resistance, particularly in receptor-targeted therapy. Future research should concentrate on identifying GPR30 expression levels, their distributions in cells and tissues, the use of GPR30 agonists/antagonists, and its use in the expansion of novel cancer treatments [76,77,304].

### 5.2. Lysophosphatidic Acid Receptor (LPAR)

On various levels, LPA has been hypothesized as a strong inducer of cancer growth. It can bind to a variety of membrane GPCRs with high affinity, and at the minimum, six GPCRs have been established as LPA receptors: LPA1–6 [12].

LPA1 plays a role in a variety of biological activities, including motility and metastasis. It causes cell transformation and has been found to be overexpressed in human breast cancer cells [80]. Similarly, cell migration, survival, and metastasis have all been demonstrated to increase when LPA2 is activated. LPA via PI3K-Akt- ERK1/2 signaling pathway was demonstrated to increase the invasive behavior of colon and ovarian malignancies [305]. There has been little research on the role of LPA3 in carcinogenesis. It has been proposed that LPA3 affects the chemotaxis of immature dendritic cells and pain levels. LPA1 inhibits human DLD1 colon cancer cells proliferation [306]. LPA2 and LPA3 have been shown to mediate the proliferation of HCT116 and LS173T cells, respectively [307], implying that LPA receptors’ ability to stimulate human colon cancer cell proliferation differs depending on cell type. These findings elucidate why the LPA1–3 receptors are regarded as prime-quality drug targets in breast cancer research.

The activation of several downstream signaling cascades occurs when LPA binds to its receptors that are linked to at least three subtypes of G proteins (G_q/11_, G_i_, and G_12/13_)). LPA receptors can activate PLC-PKC-Ca^2+^, Ras-Raf-1-MAPK, PI3K-Akt pathways [308,309]. Additionally, G_12/13_-dependent RhoA activation causes the consequences of LPA-mediated stress fiber formation and focal adhesion assembly [308,309].

Because the C-terminal of LPA2 has a special sequence that binds to class I PDZ domains, interactions with PDZ-containing proteins modulate LPA2’s effects on cellular signaling, enabling interactions with the leukemia-associated Rho guanine nucleotide exchange factor (Rho GEF) and PDZ-Rho GEF [310].

Clinical and in vitro investigations have also revealed the important role of LPA and its receptors in the tumor area, and several LPAR antagonists have been produced in response to medicinal chemistry studies. LPA1/3 competitive antagonists based on isoxazole and thiazole were originally described in 2001, with Ki16425 and Ki16198 being the most active. In a mouse model, these two drugs inhibited pancreatic cancer invasion and metastasis to the liver, lung, and brain [305,311]. BMS-986020 and SAR-100842, two LPAR antagonists, are now in clinical studies for the treatment of idiopathic pulmonary fibrosis and systemic sclerosis, respectively [312]. Additionally, cyanopyrazoles, a class of LPA1 antagonists, have been found to play a role in the regulation of inflammatory illnesses [313,314,315]. AM152, also known as BNS-986020, is another isoxazole and thiazole drug that began a phase-II clinical trial in 2015 [313,316]. Following that, whether injected, orally or subcutaneously, a small molecule LPA agonist known as Rx100 was demonstrated to effectively prevent radiation-induced mortality in mice [317]. However, no new developments have been recorded as of yet.

### 5.3. Angiotensin-II Receptor

The Ang-II peptide plays a role as a significant mediator of blood pressure and cardiovascular homeostasis by regulating the rennin-angiotensin system. There are two primary subtypes of Ang-II receptors: type-I (AT1R) and type-II (AT2R).

The AT1R is found to be overexpressed in various malignancies, including breast carcinoma cells, pancreatic adenocarcinoma cells, and hepatocarcinoma cells, as shown by in vitro studies [318]. In vivo also showed the overexpression of AT1R in ER-positive breast malignancies [319], glioblastomas [320], pancreatic ductal cancers [321], squamous cell carcinomas of the skin [322], and gastric cancers [323]. Furthermore, AT2R expression was shown to be associated with poor prognosis in astrocytomas patients [324]. These findings imply that these receptors have a role in carcinogenesis and neoangiogenesis.

Ang-II-induced AT1R signaling occurs through Gα_q/11_-phospholipases A2, C, and D pathway, thus mediates IP3/Ca^2+,^ MAPKs, tyrosine kinases (Pyk2, Src, Tyk2, and FAK), and NF-B pathway. AT1R also regulates arrestin-mediated MAPK activation and Janus kinase (JAK)/signal transducer and activator of transcription (STAT) signals in a G-protein-independent manner. These signaling pathways are known to play a major role in tumor malignancies and angiogenesis.

It has been reported that AT1R transactivates the EGFR in prostate and breast cancer cells, resulting in ERK, STAT3, and PKC phosphorylation. Furthermore, AT1R promotes endothelial cell production of VEGFR2 and angiopoietin-2 [325]. The AT1R has also been reported to have anti-apoptotic effects in microvascular endothelial cells by blocking the PI3K-Akt pathway, which results in increased survivin expression and decreased caspase-3 activity [326]. AT2R, on the other hand, suppresses endothelial cell migration and tube formation by inhibiting VEGFR2-induced Akt phosphorylation and endothelial nitrous oxide synthase [101]. EGFR autophosphorylation is also inhibited by AT2R [327,328,329]. AT2R also has a direct interaction with ErbB3, an EGFR family member [329]. A new family of AT2R-interacting proteins has recently been discovered that inhibit EGF-induced pancreatic cancer cell growth [330,331]. In conclusion, AT1R and AT2R exert opposite effects on cancer cell proliferation and angiogenesis. An in vitro research showed that AT1R antagonist lowers the expression of VEGFA [323]. Ang-II, for example, increased cell invasion and VEGFA production via AT1R in ovarian cancer cell lines. AT1R induced enhanced expression of VEGFA and VEGFR2 in lung cancer cells [332]. These data indicated that inhibiting AT1 receptor signaling could be a viable and successful cancer treatment method.

The possible involvement of angiotensin-converting enzyme inhibitors (ACEis) in anticancer research has recently piqued people’s interest [333]. The use of ACEis in experimental animal models has revealed that these medicines have a protective effect on tumor development. Captopril, an ACE that is commonly used as an antihypertensive medicine in clinical settings, dramatically decreased tumor growth, angiogenesis, and tumor diameters in xenograft models while boosting mice survival [334,335,336,337]. Furthermore, the AT1R blocker Candesartan totally decreased expression of the angiogenesis-related gene (VEGF and hypoxia-inducible transcription factor 2 (HIF-2)) and significantly reduced tumor growth, vascularization as well as lung metastases [338,339,340]. Furthermore, the specific AT1R antagonist L-158,809 dose-dependently suppressed the development of Capan-2 in a human pancreatic cancer cell line [341]. Losartan (an AT1R antagonist) administration resulted in a considerable depletion in rat C6 glioma cell proliferation and the generation of many growth factors (for example, VEGF) both in vitro and in vivo [342]. More research is necessary to assess whether AT1R blockade’s potential as a novel endocrine-targeted treatment. Because AT1R blockers have been used for hypertension therapy with no major adverse effects, we believe they could be a safe, effective, and new cancer treatment. However, very recently, a couple of cohort studies showed that long-term use of ACEis is associated with an increased risk of lung cancer, further suggesting the need for intensive research [343,344].

### 5.4. Gastrin Releasing Peptide Receptor (GRPR)

Overexpression of GRP (also known as bombesin) and its receptors have been found in a variety of cancer cells and tissues and appear to affect the growth of these neoplasms. The discovery of high-affinity GRPR in cancers led to the creation of diagnostic, radiation, and chemotherapeutic reagents.

Increased GRPR expression has been linked to the aggressiveness of neuroblastoma tumors [345]. According to Qiao et al., GRPR inhibition reduced the expression of important regulators of protein synthesis and cell metabolism by reducing the PI3K/Akt/mTOR pathway, which is typically associated with the promotion of aerobic glycolysis in cancer cells [346]. In vitro, a GRPR inhibitor reduced cell proliferation, inhibited DNA synthesis, and caused cell cycle arrest at the G2/M phase, reversing the aggressive character of the human neuroblastoma cell line BE(2)-C [347]. GRPR knockdown also inhibited neuroblastoma tumorigenicity by blocking colony formation in vitro and reducing xenograft development and liver metastasis in vivo [347,348]. In SK-N-SH cells and BE(2)-C cells, GRPR transactivated the focal adhesion kinase that activated downstream neuroblastoma tumorigenicity regulators [349]. As a result, GRP/GRPR signaling could be engaged in several stages of carcinogenesis.

Activation of GRPR has been shown to increase head and neck cancer cell invasion and proliferation by upregulating EGFR transcription and phosphorylating the downstream MAPK pathway [350,351]. Furthermore, autocrine GRP/GRPR activation can directly activate EGFR pre-ligands via Src-dependent cleavage [352] and then facilitates phosphorylation of EGFR and activation of MAPK pathway [353]. These findings imply that GRPR cross-talk with EGFR and GRPR inhibition may affect downstream signaling of EGFR by interfering with intracellular EGFR-activated mediators.

In clinical practice, a murine monoclonal antibody (2A11) has been utilized against GRP as a strong anticancer treatment and reported to decrease the incidence of lung cancer in phase I clinical trials [354]. Additionally, the injection of a new DNA vaccine that targets GRP has been suggested to reduce murine melanoma growth in vivo [355]. A recent experiment using a small-molecule inhibitor of GRP found that compound 77427 inhibited tumor cell proliferation in vitro and angiogenesis in vivo [356]. Synthetic doxorubicin–bombesin conjugates [357] and camptothecin–bombesin conjugates [358] have been shown to have a protective effect against tumor formation in animal studies. These findings suggest that GRPR-specific inhibitors have favorable consequences on tumor cell proliferation and angiogenesis, hinting that they could be used as a therapeutic tool to control tumor growth.

### 5.5. S1P Receptor

S1P mediates its biological effects in various pathophysiology, including tumor models, by activating a family of five GPCRs known as S1P1–S1P5. S1P receptors are found on various types of cells such as neurons, cardiomyocytes, and endothelial cells [359]. S1P1–3-Rs are found in nearly all tissues, S1P4-R is found mostly in lymphoid and hematopoietic tissues, whereas S1P5-R is present in the white matter of the brain and spinal cord, and spleen. These suggest that S1PR expression is tissue-specific.

S1P receptor (S1P1) activation leads to the stimulation of Gα_i_-mediated Ras-ERK, PI3K-Akt-Rac, PLC, and Rho pathways [360,361,362,363]. In colitis-associated cancer, S1P1 can interact directly with activated STAT3, enhancing tumor growth, metastasis, thus having a tumorigenic impact. S1P1 has been linked to ER-positive breast cancer tissues, possibly due to increased activation of the ERK pathway and decreased apoptosis.

Cross communication between the STAT3 and S1P-SphK-S1PR pathways has recently been discovered to play an important part in inflammation-induced carcinogenesis and tumor growth in the gut [364]. STAT3 may increase the activation of the S1P-SphK-S1PR axis, which, in turn, facilitates the sustenation of STAT3 activation in epithelial cells via a positive feedback loop [365]. These findings provide a foundation for the development of innovative sphingolipid-centric therapies and anti-inflammatory medicines to treat colorectal cancer, particularly tumors associated with inflammation.

S1P-R agonists have been utilized to treat carcinomas as effective chemotherapeutic drugs. FTY720, the S1P1,3–5-R broad specificity agonist fingolimod [366], for example, has been utilized to treat breast [367], glioblastoma [368], prostate [369], lung [370], ovarian [371], and hematological malignancies [371]. FTY720, on the other hand, is now contraindicated in patients with heart failure. More selective S1P1-R agonists in clinical studies, such as PF-04629991 [372], Ponesimod [373], CS-077 [374], and BAF312 [375], all have cardiovascular risks.

## 6. GPCRs as Cancer Targets

GPCRs are an important choice for many medicines, and their relevance in drug discovery may be gauged by the evidence that almost 60% of pharmaceuticals in development and 36% of FDA-approved commercially marketed drugs target GPCRs of humans. GPCRs are thought to be the topmost effective therapeutic targets for a variety of solid tumors as well, as many GPCRs have a role in cancer start and development and thus have the ability to directly or indirectly modify the therapeutic efficacy and survival of the patient. However, only a handful GPCRs have been effectively used to create drugs that block cancer-related signaling pathways. As more information on GPCR biology emerges, it is becoming clear that functional selectivity and “biased agonism” exist; as a result, there is less eagerness for the theory of “one medication per GPCR target” and more heed in identifying various pharmacological possibilities.

Understanding how GPCRs are activated is crucial since the method can be used to design anticancer drugs. Many plausible options for creating innovative cancer therapy strategies exist. Two methods to GPCRs focused drug development include targeting the GPCRs signaling with agonists or antagonists, as well as targeting the particular interactivity between GPCRs and their binding partners to deliver anti-neoplastic drugs or toxins to malignant cells. One example is an endocrine therapy for hormone-responsive prostate cancer that reduces testosterone levels by targeting the GnRH receptor. This method aids in the treatment of prostate cancer because encouragement of prostate cancer cell development necessitates the generation of testosterone through a signaling pathway that initiates with hypothalamic GnRH secretion [376]. An immunological technique can also be utilized to prevent endogenous agonists from interacting with a particular GPCR, such as direct vaccine injection, to provide the desired neutralizing effect. A rare example, Immunogen G17DT, is under assessment in a phase III trial to treat pancreatic cancer [377]. Similarly, GPCRs and insulin/insulin-like growth factor 1 receptors have been shown to work together to regulate a wide range of physiological activities as well as tumor formation. For example, Metformin, a diabetes treatment, hinders this cooperation and has been shown in epidemiological studies to lessen the incidence of tumors in diabetic people. The ETAR antagonists ZD4054 and atrasentan used combined with the EGFR inhibitor gefitinib and the monoclonal HER2-specific antibody trastuzumab, respectively, present a powerful capacity to repress the proliferation and invasion of cancer cells. However, ZD4054 and astrasentan have been discontinued in the clinical trials due to their several adverse effects on the patients and are no longer used as anti-tumor drugs.

Very recently, Nicholas et al. described the results from biological screens of diverse small molecules derived from the indole alkaloid yohimbine that displayed highly differential antagonistic activities against a panel of GPCRs [378]. Among them, Y7g exhibited selective antagonistic activities against vasopressin receptor 2 and oxytocin receptor suggesting that targeting GPCR by small molecules can lead to the identification of new compounds capable of interacting with distinct cancer-relevant targets.

Therefore, the pharmacological handling of various GPCRs can be an outstanding alternative to block tumorigenic signals, creating GPCR-mediated functions promising therapeutic targets in drug evolution towards novel intervention in cancer. Certain anticancer drugs and antibodies targeting GPCRs that are currently used, under trials, or GPCRs that are potential targets are listed in Table 3, Table 4 and Table 5, respectively (recently reviewed by [379,380]).

## 7. Importance of GPCRomics in Cancer

In addition to some endocrine and hormone-responsive cancers, GPCRs, although being the biggest family of approved drug targets, are rarely addressed for cancer therapy. The deficit in consideration of GPCR-targeted medicines as cancer treatments may relate, at least in part, to a lack of knowledge about GPCR expression in tumor cells and tumor environment because little prior research has been done on their expression or function. It is important to determine which GPCRs are overexpressed and what downstream signaling mechanisms are involved during carcinogenesis as a growing body of evidence connects abnormal GPCR expression and activation to a variety of cancers in humans.

Several GPCRs, for example, are overexpressed in certain tumors, and GPCR variations can enhance cancer risk. Utilizing biotechnological aassays such as GPCR-specific PCR, RNA-seq, database mining, and analysis would definitely help to describe the expression of GPCR in primary cancer cells, cancer cell lines, cells in tumor tissue, and the tumor microenvironment.

For example, MC1R polymorphisms were shown to be linked with an increased threat to skin cancer [65]. Additionally, aberrant activation of GPCRs by high levels of ligands like LPA, S1P, and chemokines was shown to be involved in cell transformation, proliferation, angiogenesis, metastasis, and drug resistance. Contrarily, some GPCRs, such as the orexin receptor OX1R was shown to modulate a pro-apoptotic action in different cancer cells [387]. Similarly, increasingly expressed GPCRs in cancer cells (for example, GPRC5A in PDAC and colon cancer cells and GPR68 in PDAC CAFs), according to Paul et al., might add to the malignant phenotype, act as biomarkers, and/or constitute new drug targets for cancer therapy [388,389].

Some cancer cell types might have a “GPCR signature”, suggesting that one or more GPCRs could be used as biomarkers and/or drug targets in these tumors. Assessment of the GPCRs selectively expressed in cancer cells, including recognition of GPCR protein expression, signaling, and functional activities, will be required for therapeutic utility. Initial research suggests that at least some of the GPCRs Paul et al. discovered are active in cancer cells (for example, GPR161 in breast cancer [390] and GPRC5A in pancreatic cancer [391]) and the micro-environment (e.g., GPR68 [389]). This demonstrates that various GPCRs are selectively overexpressed in distinct cancer types, implying that each cancer type might have its own “GPCR-ome”. Some GPCRs (e.g., CD97 and GPR56) are widely expressed in normal tissue and cells, as well as in cancer [392]. The recognition of “driver mutations”, which are divided throughout various forms of cancer and anticipated to be sensitive to molecularly targeted treatments, has become a major priority for fundamental and clinical researchers in recent years. Simultaneously, personalized (precision) medicine approaches based on genomic analysis to identify such driver mutations have grown in popularity. Aside from the existence of these “illicitly” produced receptors, it seems that there is no such GPCR profile that is shared by cancer cells of various types. GPCRomic studies should be undertaken to define the expression of endoGPCRs that are stimulated by endogenous molecules, including hormones, neurotransmitters, and metabolites in diverse types of cancer cells.

## 8. Concluding Remarks and Future Perspectives

GPCRs activation induces a variety of signaling cascades along with the activation of second messengers, GEFs, Ras and Rho GTPases, MAP kinases, PI3Ks, and various downstream cytosolic and nuclear targets that affect normal cell functions such as growth, survival, differentiation, and migration. Cancer cells are also able to utilize these pathways for their benefits, such as tumor growth enhancement, promotion of angiogenesis, invasion, and metastasis, and entry into the immune system. Since the drug resistance against cancer is frequent, potential therapy against cancer can be developed by targeting either GPCRs or selective downstream signaling molecules. Despite the enormous potential of GPCRs as the most important therapeutic targets, their importance in and as cancer targets are under-utilized, with only a few anticancer drugs that exploit GPCRs and their signaling molecules now being employed clinically. The evolution of novel targets and innovative pharmaceutical techniques for cancer patients’ treatment will most likely be aided by continuing a more thorough investigation to completely comprehend the biological activities and related molecular mechanisms of the various GPCRs behind tumor progression and metastasis. Extensive work will be necessary to specifically target GPCRs with selective functions that would minimize the risk of side effects. Additionally, the network maps: GPCR interactome, can be identified that would connect several GPCR-dependent signaling events interacting with other signaling pathways manipulating which could lead to future investigations regarding the biophysical potential of these receptors in the design of cancer-tailored novel therapeutics for more focused clinical practice.

In conclusion, this review provides a wide perspective of the biological role generated by GPCRs in carcinogenesis and anticipates that improved knowledge of GPCRs molecular pharmacology, along with a profusion of modern high-throughput screening methods, will likely lead to the creation of an altogether new generation of GPCR-based therapies, resulting in significant clinical improvements for cancer patients.

## Figures and Tables

**Table 1:** Selected G-protein-coupled receptors, ligands, and signaling pathways involved in cancer. Adapted and modified from Bar-Shavit et al. [25].

Receptor/s	Ligand/s	Pathway/s	Cancer Type
Lysophosphatidic acid receptors LPA1-6)	LPA	Rho-dependent pathways [26,27]	Colon cancer [28,29] Ovarian cancer [30] Prostate cancer [31] HNSCC [32] Breast cancer
		β-cantenin stabilization [33,34]	
		Kruppel-like factor 5 [35]	
Protease-activated receptors (PAR1&2) LPA	Thrombin, trypsin, or TFLLRN (PAR1) or SLIGKV (PAR2) Lysophosphatidic acid (G_αq_)	Hippo/YAP pathways via activation of G_α12/13_-coupled receptors or G_αq_. Inhibition of Hippo pathway (via the inhibition of Lats1/2 kinases) [36]	Breast cancer [37] Colon cancer [38] HNSCC [39] Prostate cancer
Frizzled (Fz) PAR1 Parathyroid receptor1 (PTHR1)	Wnt 3A (canonical pathway)	Canonical Wnt signaling stabilization of β-catenin and its transcription activity [40]	Colon cancer [40,41] Lung cancer [42,43] Breast, gastric, and thyroid cancers and melanoma [43,44] Prostate cancer [45]
	Thrombin or TFLLRN		
	PTH		
Chemokine receptor (CXCR4)	CXCL12, SDF-1	PI3K, Akt, Src, PIP2, IP3, Ras, Raf, ERK1/2, PLC, JNK [46]	Melanoma Pancreatic cancer Prostate cancer Breast cancer Ovarian and thyroid cancers HNSCC [31] Lung cancer Neuroblastoma and kidney cancer
Endothelin receptors (ETAR and ETBR)	Endothelin 1–3 (ET-1, ET-2, ET-3)	c-Src/cross-talk with EGFR	Ovarian cancer Colon and prostate cancers [47] Breast cancers [48] Endometrial cancer [47] Rhabdomyosarcoma
		β-arrestin1 or 2 PDZRhoGEF and Rho A, C	
		β-catenin stabilization [49,50]	
Prostaglandin receptors (PE2, PE4)	PGE2	Cyclooxygenase pathway, PI3K (coupling to G_αi_) [51,52,53]	HNSCC [32] Breast cancer [31] Lung cancer [31] Prostate cancer [31] Colon cancer [54]
Bradykinin receptor Type 1 and 2 (B1R, B2R)	Kinins	G_αq_ and cross-talk with EGFR Ras, Raf, ERK	Chondrosarcoma HNSCC [32] Prostate cancer
Sphingosine 1- phosphate receptor (S1PR)	S1P	Ras-ERK, PI3K/-Akt/-Rac, Rho, STAT3 (coupling to G_αi_) [55,56]	Glioma [57,58,59] Breast and prostate cancers Ovarian cancers
Angiotensin II type 1 receptor	Angiotensin II	TNF-α, ERK1/2, NF-κB, STAT [60,61]	Gastric cancer [62] Prostate cancer [31]
Gastrin-releasing peptide receptor	Gastrin-releasing peptide	NF-κB, p38^MAPK^, PI3K/-Akt [63,64]	HNSCC Lung and pancreatic cancers Prostate cancer

**Table 3:** Currently used FDA-approved drugs and antibodies against different cancers.

Drugs	Receptor	Cancer Types	Year of Approval
Cabergoline	Dopamine receptor D1 (DRD1)	Neuroendocrine tumors, pituitary tumors	1996
Lanreotide	Somatostatin receptor (SSTR)	Pancreatic cancer	2007
Degarelix	GnRH	Prostate cancer	2008
Vismodegib (Erivedge)	SMO	Locally advanced and metastatic basal cell carcinoma	2012
Sonidegib (Odomzo)	SMO	Locally advanced and metastatic basal cell carcinoma	2015
Mogamulizumab	CCR4	T-cell lymphoma	2018

**Table 4:** Anti-GPCRs drugs and antibodies under clinical trials.

Cancer	Inhibitor	Type of Molecule	Receptor	Phase	Sponsor/s
Head and neck cancer	GDC-0449 (Vismodegib)	Small molecule	SMO	Phase II	Sue Yom in collaboration with Genentech, Inc.
Ovarian cancer	GDC-0449 (Vismodegib)	Small molecule	SMO	Phase II	Genentech, Inc.
	Propranolol (beta-blockers)	Small molecule	Beta-adrenergic receptor	Phase I	Washington University School of Medicine
Pancreatic cancer	CCX872 (OMP-18R5)	Small molecule	CCR2	Phase I	ChemoCentryx
	Vantictumab	Antibodies	Frizzled receptor FZD7	Phase I (combine with nab-paclitaxel and gemcitabine)	OncoMed Pharmaceuticals, Inc.
	G17DT	Immunogen	Cholescystokinin-2 receptor	Phase III	Cancer Advances Inc.
Multiple myeloma	BMS-936564	Antibodies	CXCR4	Phase I	Bristol-Myers Squibb
Melanoma	Plozalizumab	Humanized monoclonal antibody	CCR2	Phase I	Millennium Pharmaceuticals, Inc
Adult T-cell leukemia and lymphoma	KW-0761 (Mogamulizumab)	Antibodies	CCR4	Phase II	Kyowa Kakko Kirin
Metastatic breast cancer	OMP-18R5 (Vantictumab)	Antibodies	Frizzled receptors (FZD1, 2, 5, 7, 8)	Phase I (combined with paclitaxel)	OncoMed Pharmaceuticals, Inc.
	Beta-blockers	Small molecule	Beta-adrenergic receptor	Phase II	Columbia University
Non-small cell lung carcinoma	OMP-18R5 (Vantictumab)	Antibodies	Frizzled receptors (FZD1, 2, 5, 7, 8)	Phase I (combined with docetaxel)	OncoMed Pharmaceuticals, Inc.
Advanced solid tumors	AAT-007	Small molecule	Prostagladin E2 receptor (EP4)	Phase II	University of Maryland
Advanced or metastatic cancer	LY-2624587	Antibodies	CXCR4	Phase I	Eli Lilly and company

**Table 5:** Potential GPCR targets for cancer therapy.

Cancer type	Receptor	Ligand	Experiment Model/s	Results	References
Colon cancer	Formylpeptide receptor-2 (FPR2)	F2L	Human colon cancer cell lines	Knockdown of FPR2 from colon cancer lines resulted in reduced tumorigenicity.	[381,382]
Pancreatic cancer	G_α_-coupled beta-adrenergic receptor	Beta-blocker	Hamsters, transgenic mice	Blockage of beta-adrenergic signaling by beta-blocker prevented pancreatic cancer in mice.	[383]
Prostate cancer	AT1R	Ang II	LNCap and PC3 cells	Inhibition of growth factor signaling was observed in LNCaP and PC3 cell lines.	[384]
	GPR160	Instead of cognate ligands, lentivirus-mediated shRNA system was used to suppress GPR160 transcription.	PC3, LNCaP, DU145, and 22Rv1 cells	Treatment of PC3 cells with GPR160-targeting shRNA lentiviruses resulted in cell apoptosis and growth arrest.	[385]
Head and neck cancer	CXCR7, an atypical chemokine receptor also referred to as ACKR3	Single variable domains of a highly selective immunoglobulin were used.	HNSCC cells	Immunoglobin therapy inhibited CXCR7-expressing head and neck cancer xenografted cells in nude mice.	[386]

## Data Availability

Not applicable.

## Abbreviations

GPCR	G protein-coupled receptor
PAR	Protease-activated receptor
LPA	Lysophosphatidic acid
WNT	Wingless and Int-1
7-TM	Seven transmembrane
EL	Extracellular loop
LGRs	Leucine-rich repeat-containing receptors
PLC	PhopholipaseC
PIP2	Phosphatidylinositol 4,5-bisphosphate
DAG	Diacylgylcerol
IP3	Inositol 1,4,5-triphosphate
PKA	cAMP-dependent kinase
PKC	Protein kinase C
PKG	cGMP-dependent kinase
CAMKs	Calcium-calmodulin regulated kinases
GIRK	G-protein-gated inwardly rectifying potassium channel
PI3K	Phosphoinositide 3-kinase
S1P	Sphingosine-1-phosphate
MAPK	Mitogen-activated protein kinase
GRKs	G-protein receptor kinases
Ang-II	Angiotensin II
BK	Bradykinin
AT1R	Angiotensin-II type-1 receptor
HRH1	Human histamine receptor H1
GnRH	Gonadotropin-releasing hormone
TSHR	Thyroid-stimulating hormone receptor
LHCGR	Luteinizing hormone receptor
FSHR	Follicle-stimulating hormone receptor
SMO	Smoothened
PTCH	Patched
NSCLC	Squamous non-small cell lung cancer
GR	Glutamate receptors
AVPR	Arginine vasopressin receptor
MC2R	Melanocortin 2 receptor
NK	Natural killer
SRF	Serum response factor
AP-1	Activating protein 1
PTH	Parathyroid hormone
ETAR	Endothelin A receptor
MMPs	Matrix metalloproteases
ECM	Extracellular matrix
VEGF	Vascular endothelial growth factor
PGE2	Prostaglandin E2
COX	Cyclooxygenases
NSAIDs	Nonsteroidal anti-inflammatory medications
TAMs	Tumor-associated macrophages
DLBCL	Diffuse large B cell lymphoma
Fz	Frizzled
APC	Adenomatis polyposis coli
GSK	Glycogen synthase kinase
CK1	Casein kinase1
LRP	Lipoprotein-related protein
ET	Endothelin receptor
EMT	Extracellular matrix
PCP	Planar cell polarity
ROCK	Rho-associated kinase
YAP	Yes-associated protein
TAZ	Transcriptional coactivator with PDZ-binding motif
PH	Pleckstrin homology
mAChRs	Muscarinic receptors
EFGR	Epidermal growth factor receptor
PDGF	Platelet-derived growth factor receptor
GRP	Gastrin releasing peptide
BN	Bombesin
HB-EGF	Heparin-bound EGF
ER	Estrogen receptor
STAT	Signal transducer and activator of transcription
ACEis	Angiotensin-converting enzyme inhibitors
HIF-2	Hypoxia-inducible transcription factor 2
OXR	Orexin receptor
